# [Expression of Concern] TIP60 governs the auto-ubiquitination of UHRF1 through USP7 dissociation from the UHRF1/USP7 complex

**DOI:** 10.3892/ijo.2026.5908

**Published:** 2026-06-24

**Authors:** Tanveer Ahmad, Waseem Ashraf, Abdulkhaleg Ibrahim, Liliyana Zaayter, Christian D. Muller, Ali Hamiche, Yves Mély, Christian Bronner, Marc Mousli

Int J Oncol 59: 89, 2021; DOI: 10.3892/ijo.2021.5269

Following the publication of this paper, it was drawn to the Editor's attention by a concerned reader that the GAPDH control western blots shown in Fig. 5A on p. 9 for the experiments without MG132 treatment ('- MMGM132') were strikingly similar to the GAPDH control western blots shown in Fig. 5D, albeit with some horizontal and vertical resizing of the bands. In addition, an independent analysis of the data in this paper revealed that the flow cytometric plots for the TIP60-transfected cells experiment in Fig. 8B on p. 12 were also strikingly similar to the plots shown to represent the TIP60-eGFP Positive cells experiment in Fig. 8E.

The authors have been contacted by the Editorial Office to offer an explanation for the apparent re-use of the abovementioned data in this paper, and we are awaiting their response. Owing to the fact that the Editorial Office has been made aware of potential issues surrounding the scientific integrity of this paper, we are issuing an Expression of Concern to notify readers of this potential problem while the Editorial Office continues to investigate this matter further.

